# Translation approaches to support systemic anti-cancer therapy consent for individuals with limited English proficiency

**DOI:** 10.1007/s00520-026-10464-w

**Published:** 2026-03-13

**Authors:** Stephen P. Hibbs, Rumman Nizam, Ubaid Tanzim, Laura Aiken, Sabrina Habib, Sam Hodgson, Jill Williams, Adam Januszewski, Andrew Hantel, Mohammed Salah Uddin, Talia Isaacs, Bettina Bajaj, Olivia Cockburn, Amin Islam, Guy Pratt, Federico M. Federici

**Affiliations:** 1 Wolfson Institute of Population Health, Queen Mary University of London, Yvonne Carter Building, 58 Turner St., London, E1 2AB UK; 2 George’s University Hospitals NHS Trust, London, UK; 3 University College London Hospitals NHS Foundation Trust, London, UK; 4 Department of Clinical Haematology, Barts Health NHS Trust, London, UK; 5 Barts Cancer Centre, Barts Health NHS Trust, London, UK; 6 Myeloma UK, Edinburgh, UK; 7 Department of Medical Oncology, Dana-Farber Cancer Institute, Boston, MA USA; 8 Center for Bioethics, Harvard Medical School, Boston, MA USA; 9 National Register of Public Service Interpreters (NRPSI), Chartered Institute of Linguists, London, UK; 10 Middlesex University, London, UK; 11 UCL Institute of Education, University College London, London, UK; 12 Centre for Translation Studies (CenTraS), University College London, London, UK; 13 Department of Haematology, Mid & South Essex University Hospital NHS Foundation Trust, Southend, UK; 14 Institute of Health Sciences Education, Queen Mary University of London, London, UK; 15 Cancer Research UK Clinical Trials Unit, University of Birmingham, Birmingham, UK

**Keywords:** Treatment consent, Health equity, LEP health literacy, Risk comprehension, Machine-translated patient brochures, Interpreter-mediated oncological consultations

## Abstract

**Purpose:**

Limited English proficiency (LEP) is associated with poor cancer outcomes, and written information about systemic anticancer treatment (SACT) is often provided in English only. Unsupervised machine translation of medical information is common, but its effectiveness and accuracy are unclear. This study aimed to (a) compare professional and machine translation of written SACT information and (b) investigate whether providing a bilingual SACT consent form altered comprehension of key information during an interpreted SACT consent consultation.

**Methods:**

This randomised study included healthy, Bengali- or Sylheti-speaking adults with LEP across London, UK. Participants were randomised twice. First, they were allocated to either a machine translation or professional (human) translation of an English language SACT information booklet. Second, 1–2 weeks later, participants underwent a simulated SACT consent consultation with a doctor and interpreter, and were allocated to either a conventional English-only SACT consent form or a bilingual English–Bengali consent form.

**Results:**

After reading the translated booklet, 19/121 participants (15.7%) met the primary outcome of understanding treatment intent, with no difference by translation type (multivariate OR = 0.99, *p* = 0.99). The machine-translated booklet contained 11 meaning-changing errors, compared to 1 in the professional translation. Of 91 participants completing the consent consultation, randomisation to the bilingual translated SACT form was associated with higher odds of understanding treatment intent (multivariate OR = 3.73, *p* = 0.01).

**Conclusion:**

In this study, a bilingual English–Bengali consent form improved comprehension amongst LEP participants and may hold value in other language-discordant settings. Unsupervised machine translation of a patient information booklet introduced more errors than professional translation, but most individuals receiving either translation type did not comprehend crucial information; other information formats should be explored.

**Trial registration:**

University College London Research Ethics Committee approved this study as a low-risk application on 28 October 2024 at 15:15, reference number: AH/2024/52-6625/003. The statistical analysis plan of the study was registered in the Open Science Framework, https://osf.io/axg5d.

**Supplementary information:**

The online version contains supplementary material available at 10.1007/s00520-026-10464-w.

## Introduction

Language discordance between patients and clinicians is common. In the UK, over one million people report limited English proficiency (LEP) [1]. LEP is a key social determinant of cancer incidence, prevention and control [2–4], which may reflect both the association of LEP status with other markers of social disadvantage, and the direct effects of language discordance on health communication [2, 5]. Poor healthcare communication with LEP patients leads to increased healthcare costs [1] because of delayed diagnoses and increased healthcare utilisation, as demonstrated among patients with multiple myeloma [6]. The absence of suitable translation and interpreting services also decreases treatment adherence among LEP populations with cancer [7, 8], reduces their participation in oncological clinical trials [9, 10] and limits their participation in research [10].

Language discordance creates additional challenges when obtaining informed consent for systemic anticancer therapy (SACT)—a complex, risk-laden and technical therapeutic approach within which understanding treatment intent is critical [11]. Whereas guidelines exist for translation of written participant information and consent forms in a clinical trial setting [12], there is no such guidance for routine oncology practice. Conventional documentation in routine oncology consultations, including written information material and structured SACT consent forms, is often provided in English only [13], with reliance upon verbal interpretation for communication with LEP patients.

Unsupervised machine translation, defined as the use of computerised algorithms to generate translations without human validation, is widely used by healthcare professionals, health organisations and patient charities [14], such as through integration of the neural machine translation tool Google Translate into accessibility software (such as ReciteMe) [15], online triage tools and patient information apps. For high-resource languages with a large linguistic corpus to train machine translation tools, machine translation has shown promise. For example, a recent study showed high accuracy in translating paediatric instructions into Spanish using Chat-GPT (Open AI), but emphasised that “[t]ranslation performance is language specific […] results cannot be generalized to other languages” [16].

These technologies increase access to information resources for LEP patients in their preferred language [16, 17], but their use must assess clinical risks in oncological settings [18]. Best practices are for machine translation outputs to be reviewed by professional translators [19], but this is often omitted in real-world situations [14]. Furthermore, existing research assesses the accuracy of machine translations, rather than their impact on translation users [17, 20]. Specifically, the impact of different translation approaches on comprehension in a SACT consent context has not been formally evaluated.

To address these unanswered questions, our study had two co-primary aims: (1) to compare the effectiveness and quality of professionally translated and unsupervised machine-translated versions of SACT patient information in a low-resource language (i.e. a language with a limited linguistic corpus to train machine translation tools), and (2) to assess the impact of providing a professionally translated English–Bengali bilingual consent form on both objective comprehension and self-rated understanding during an interpreted SACT consent consultation. We chose myeloma as a case study, given its relative unfamiliarity and non-curative treatment intent. We studied Bengali- and Sylheti-speaking communities in the UK because of their high rates of LEP and poorer health outcomes compared to the wider UK population [21].

## Methods

### Study design

We conducted a study involving two randomised components: a Booklet Component and a simulated Consent Component. Participants were allocated to intervention groups on a 1:1 ratio for both components. Bengali- and Sylheti-speaking patients and clinicians contributed to the design, conduct and analysis of the study as detailed in Supplementary Methods. This study followed the Consolidated Standards of Reporting Trials (CONSORT) reporting guidelines [22].

### Participants and setting

Participants were recruited from Bengali- and Sylheti-speaking community groups within East and North London, through a combination of directed outreach by community leaders, social media posts and visits to existing community groups by the research team. Due to this recruitment approach, we do not know how many people considered the invitation but chose not to participate. Eligible individuals were offered a £20 gift voucher for their participation in each component.

Adult volunteers were eligible to participate if they met three criteria: (a) self-identifying as preferring to receive medical information in Bengali or Sylheti instead of English, (b) able to read written Bengali and (c) able to provide written consent. For context, Sylheti is a minoritised and politically unrecognised language of Bangladesh which no longer has its own standard script and is written in a Bengali script [23]. Sylheti is the most common dialect of Bengali in the UK and is predominantly spoken rather than written; many speakers of Sylheti read written Bengali [24]. Ethical approval was given by the University College London Research Ethics Committee. Potential participants received study information through two modalities: a Bengali-language video explanation alongside a written participant information professionally translated into Bengali; written consent was collected from all participants.

### Intervention and comparator

The two components of the study were separated by 1–2 weeks, aiming to simulate the provision of information to a newly diagnosed myeloma patient prior to commencing daratumumab, bortezomib, thalidomide and dexamethasone (current first-line treatment for transplant-eligible patients in the UK) [25]. For both components, participants were asked to imagine that they were supporting a friend or family member with a new diagnosis of myeloma. Full details of interventions are provided in Supplementary Methods.

#### Booklet component

Each participant was given a Bengali translation of an English booklet (“Easy Read: How is myeloma treated?”, *Myeloma UK*) [26]. The booklet is designed to provide accessible and easy-to-read information, and is written in accordance with the European Easy-to-Read standard [27]. An unsupervised neural machine translation (NMT) was generated using Google Translate; this NMT tool was chosen for two reasons. First, NMT tools are currently superior to large language models in producing domain-adapted and context-aware translation outputs in Bengali [28]. Second, many UK healthcare providers and cancer charities use Recite Me [15] to enable patients to read their patient information in non-English languages including Bengali; this accessibility tool uses Google Translate to create translations. Participants were randomised to provision of a professional (human) translation or an unsupervised machine translation. Immediately after reading their allocated booklet, participants were asked to complete the Booklet Comprehension Tool. Both Comprehension Tools were validated in English, then translated into Bengali and further checked (details provided in the Supplementary Methods).

#### Consent component

One to two weeks later, participants undertook a simulated SACT consent consultation with a haematologist and professional interpreter, structured using the nationally approved generic SACT consent form [13]. Participants were randomised to receive either an English-only consent form or a newly created, professionally translated, interlinear, bilingual Bengali–English consent form. Interlinear translations maintain proximity of source and target languages, allowing use by readers of both languages. Immediately after the simulated consent consultation, participants were asked to complete the Consent Comprehension Tool.

### Outcomes

We chose a primary outcome of the proportion of participants understanding treatment intent: specifically, that the myeloma SACT regimen would not cure myeloma but instead aims to increase lifespan and quality of life. This information is explicitly included in the Easy Read booklet that was translated for the Booklet Component, and in both written and verbal form during the Consent Component. We created, piloted and refined two comprehension assessment tools for each component to assess the primary outcome, other critical points of SACT understanding within each component (Total Comprehension Score) and self-rated confidence in understanding (Comprehension Tools and outcome definitions are detailed in Supplementary Methods).

For the Bengali translations used in the Booklet Component, an additional secondary outcome was blinded independent assessment of translation quality using the Chartered Institute of Linguists (CIoL) criteria for the Diploma in Translation. Two overarching criteria were assessed: Comprehension (assessing semantic accuracy, fidelity of localisation of names, dates, figures and lexis) and Language Quality (assessing grammar, syntax, cohesion, structure, orthography, punctuation and register). “Very serious errors” are defined by CIoL as errors that completely change meaning or make translated texts unusable unless retranslated or heavily revised (also defined as “critical errors”) [29, 30].

Other than negative impacts on trust or comprehension arising from potential mistranslation, we did not anticipate direct harms arising from our interventions.

### Sample size, randomisation and blinding

This was an exploratory study with no pre-existing data on expected rates of SACT comprehension within this population, and accordingly, we were unable to make a formal sample size calculation. We aimed to maximise the sample size within the constraints of available time, funding and capacity of community partners.

We used the blocked randomisation list tool at www.sealedenvelope.comto generate a randomisation sequence in block sizes of 4 and 6 [31]. Sealed envelopes in participant order containing the randomisation were created by a researcher who was not otherwise involved in the study; no other team members had access to the randomisation sequence. Sealed envelopes were given to participants following informed consent and only unsealed by participants after enrolment. To maintain balanced allocation in the Consent Component randomisation, separate randomisation sequences were generated for each Booklet Component group: one for participants who received the machine-translated booklet and another for those who received the professionally translated booklet.

All participants were blinded to study interventions and outcomes. Independent assessors of translated information booklets were blinded to translation type. Doctors and interpreters involved in the Consent Component were blinded to study outcomes and items of information tested in comprehension tools. Researchers marking comprehension tools were blinded to group allocation.

### Statistical methods

The association of randomisation with outcomes was assessed using both univariate and multivariate logistic regression models adjusted for self-reported age, gender, highest level of education, self-rated prior knowledge of myeloma and English language proficiency assessed over multiple domains.

For the Booklet Component and Comprehension Component primary outcomes, which are binary, we assessed associations using binomial generalised linear models. For the (ordinal) secondary outcomes in both phases, we examined associations using ordinal logistic regression models, specifically with cumulative link models from the R package “ordinal”. Analyses were completed using R version 4.5.1.

All participants enrolled for the Booklet Component were included for analyses, regardless of whether they proceeded to participate in the Consent Component or not. If an individual missed a question in either comprehension tool, this was marked as incorrect. If an individual did not complete any of their comprehension tools, they were not included in analysis.

## Results

**Fig. 1:**
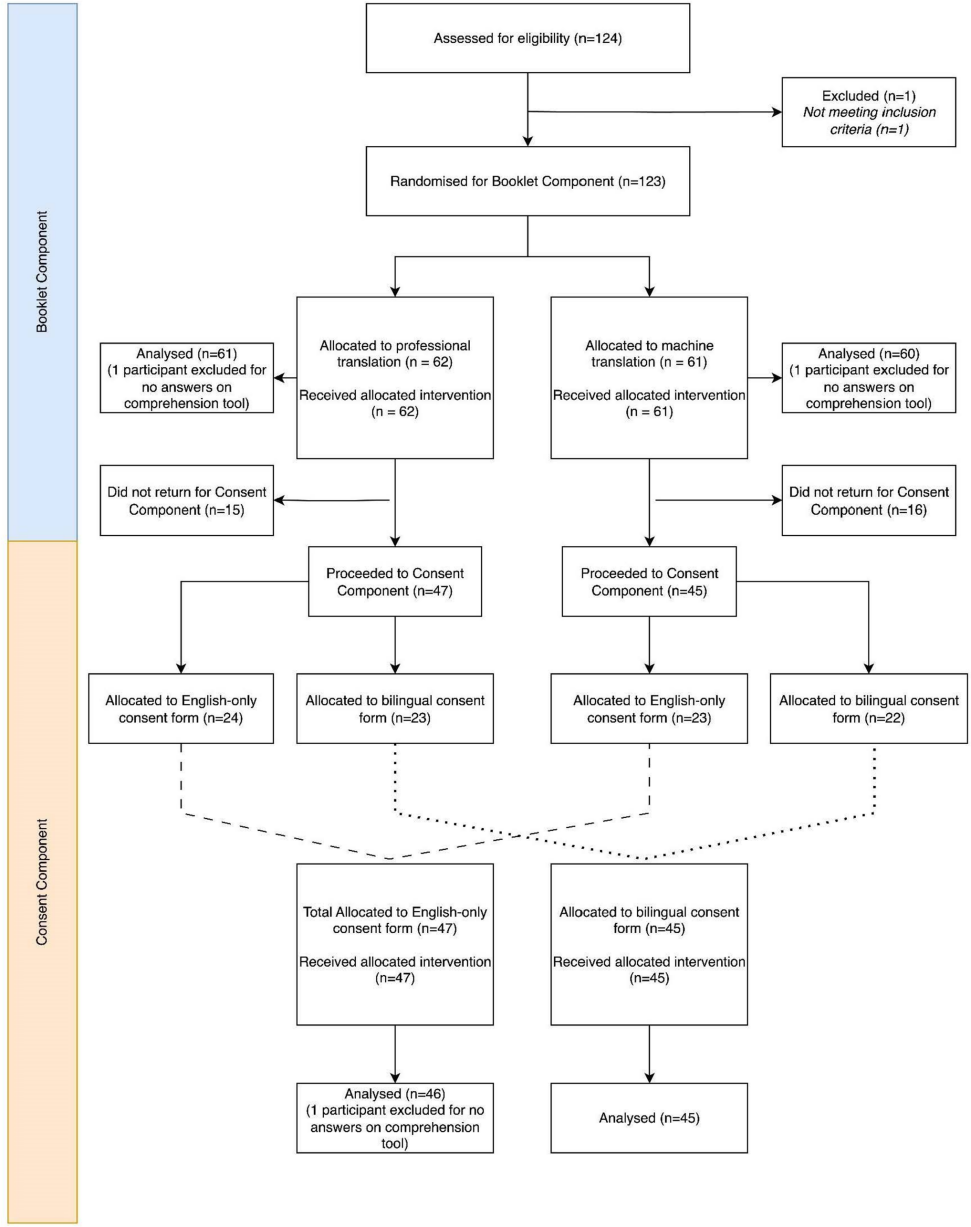
CONSORT flow diagram showing movement of participants through the study

Between 23rd April and 8th May 2025, 123 individuals were recruited and randomised during the Booklet Component, of whom 92 proceeded to the Consent Component. All participants received their allocated intervention. Flow of participants through the study is summarised in Fig. 1.

### Translated information booklet component

123 individuals were recruited and randomised for the booklet component, of whom 121 completed at least part of their comprehension tool and were included in the analysis. Of the 121 participants analysed, 42.1% were female, the majority were between the ages of 45–64 and 76% rated their ability to use English as either “Fair”, “Poor” or “Very Poor” (Supplementary Table 1 reports full demographic data).

In total, 19/121 (15.7%) of participants in the Booklet Component achieved the primary outcome of correctly understanding treatment intent, defined as correctly answering Question 12 (“What is the main goal of the treatment given to treat myeloma?”) and Question 13 (“The medicines for myeloma can cure the disease completely. True or False?”). Of 61 participants who read the machine translation, 15 (24.6%) answered Question 12 correctly, 24 (39.4%) answered Question 13 correctly and 10 (16.4%) answered both questions correctly. Of the 60 participants who read the professional translation, 14 (23.3%) answered Question 12 correctly, 29 (48.3%) answered Question 13 correctly and 9 (15%) answered both questions correctly.

Randomisation to professional or machine translations was not associated with the primary outcome (univariate OR = 1.15, 95% CI 0.43–3.07, *p* = 0.77; multivariate OR = 0.99, 95% CI = 0.33–2.99, *p* = 0.99) (Supplementary Table 2, Supplementary Fig. 1), nor any secondary outcomes (Total Comprehension Score, confidence in explaining myeloma to a family member, or perceived clarity of language; multivariate *p* = 0.46, *p* = 0.13, p = 0.74, respectively).

Independent translation assessment scored the machine-translated booklet 51/100 (“fail” by CIoL criteria) and the professional translation 73/100 (“pass” by CIoL criteria). The professional translation introduced 1 critical error and 4 accuracy issues, compared to 11 critical errors and 19 accuracy issues introduced by the machine translation. The independent assessment of the machine translation summarised that “A reasonable level of comprehension was demonstrated throughout the document. However, in several instances, there were serious issues with comprehension, resulting in text that is completely inaccurate”. Mistranslations in the machine translation included: “The medicine will help you to: Kill as many myeloma cells as possible”, as instructing the patient to kill as many myeloma cells as possible. Full independent assessment of both translations is reported in Supplementary Results.

### Simulated consent component

Demographic characteristics of participants proceeding to the Consent Component were similar to the Booklet Component and are detailed in Supplementary Table 1. Amongst 91 participants completing the simulated consent exercise, 27/45 (60.0%) of individuals randomised to the bilingual consent form group correctly understood treatment intent as non-curative, compared to 16/46 (34.8%) of individuals randomised to the English-only consent form group.

**Fig. 2:**
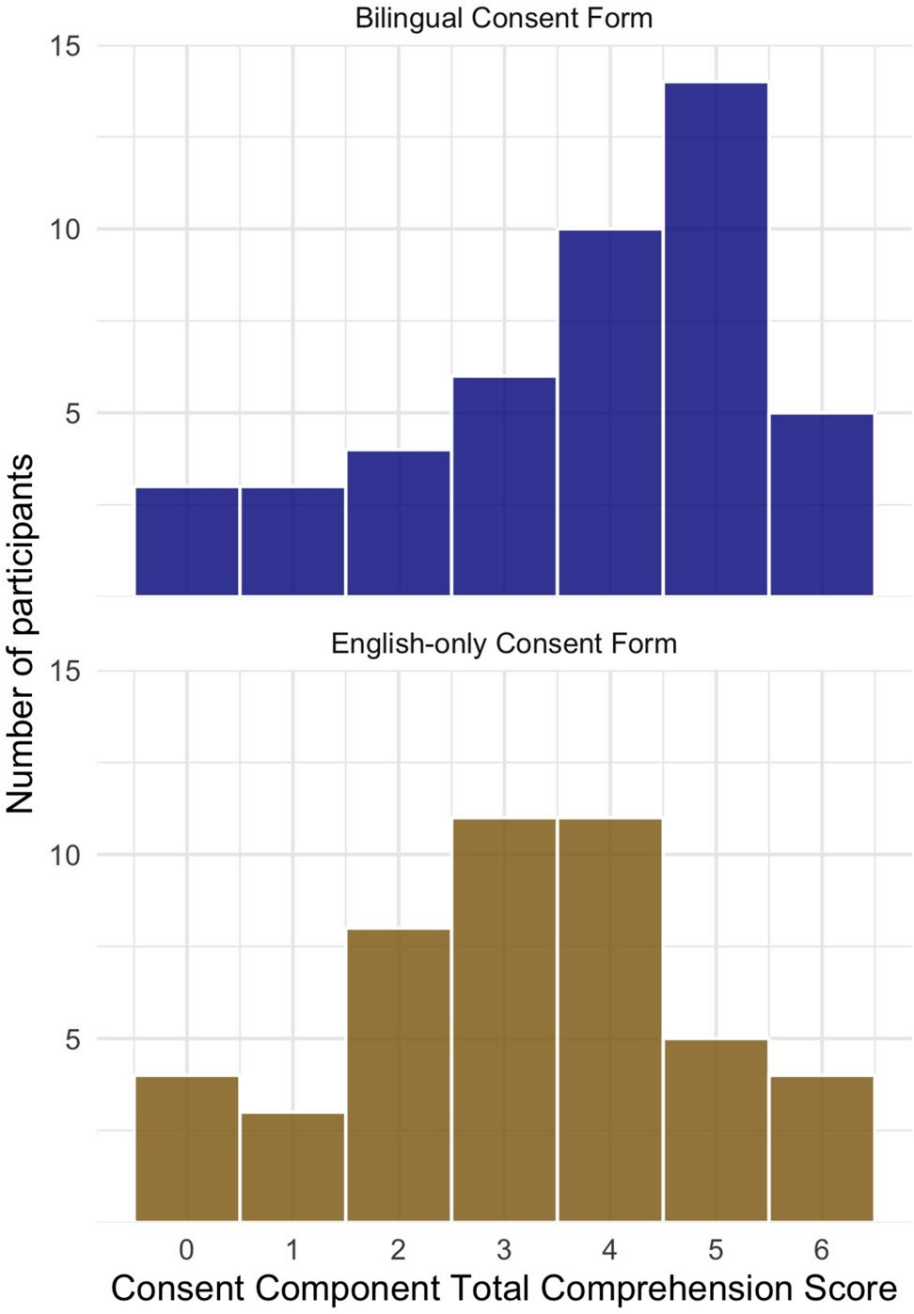
Consent Component primary outcome associations with demographic variables and randomisation status

Compared to those receiving an English-only consent form, participants randomised to receive a bilingual consent form were more likely to meet the primary outcome measure of correct understanding of SACT treatment intent (multivariate OR = 3.73, 95% CI = 1.38–10.14, *p* = 0.0097; associated univariate OR = 2.81, 95% CI = 1.20–6.59, *p *= 0.017) (Fig. 2, Supplementary Table 2).

**Fig. 3:**
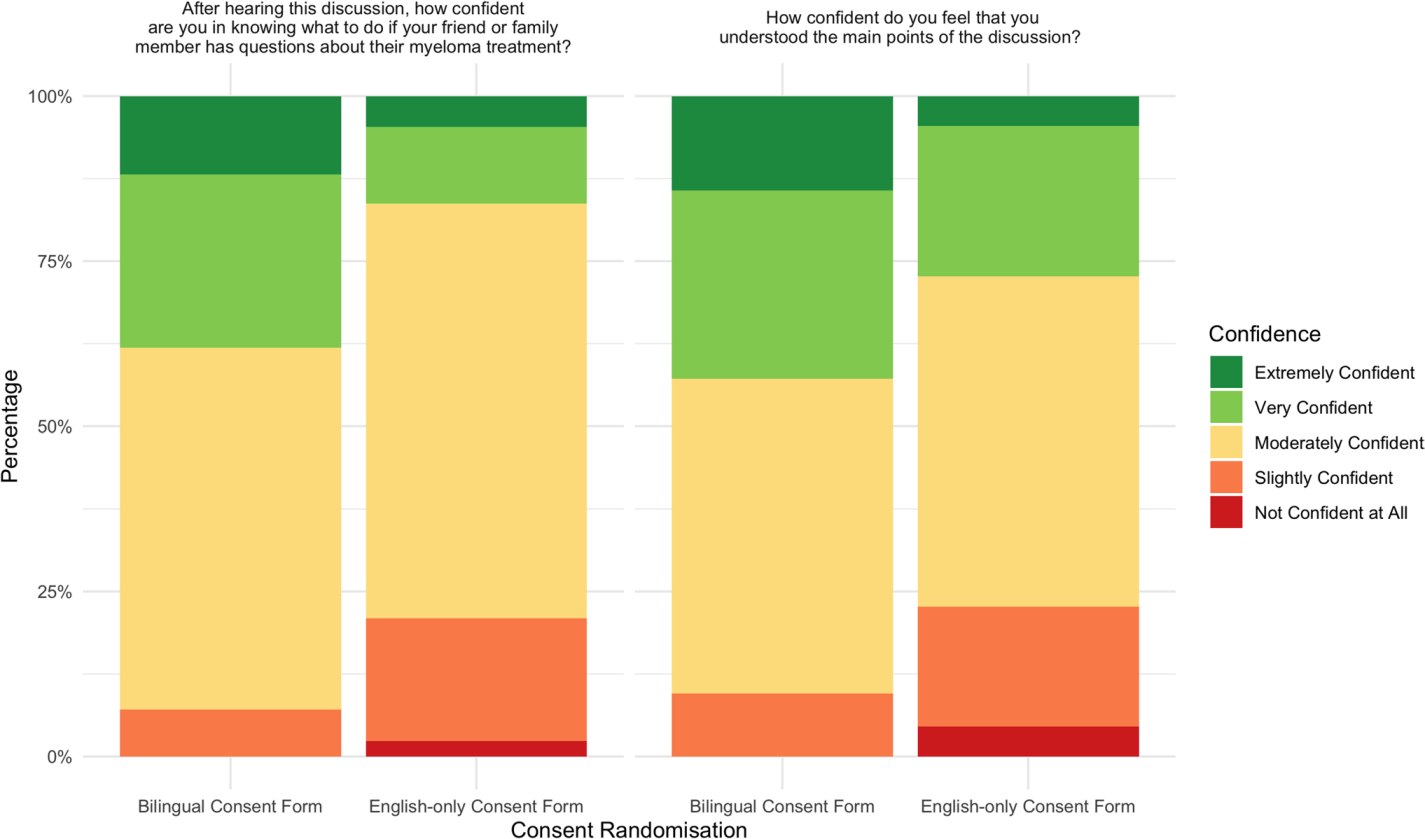
Self-rated confidence amongst Consent Component participants stratified by the randomisation group

Randomisation to the bilingual consent form was also associated with higher Likert-type scale scores (on scale 1–5) for confidence in knowing what to do if a friend or family member had questions about myeloma treatment (multivariate beta = 0.42, 95% CI = 0.02–0.82, *p* = 0.04) and confidence in having understood the main points of the consent consultation (multivariate beta = 0.51, 95% CI = 0.05–0.97, *p* = 0.03) (Spread of Likert responses shown in Fig. 3). Out of a maximum Total Comprehension Score of 6, participants randomised to receive the bilingual consent form had a median score of 4 compared to 3 for those receiving the English-only consent form (Supplementary Fig. 2). This difference was statistically significant in univariate regression models (beta = 0.74, 95% CI = 0.007–1.49, *p* = 0.048) but not multivariate (beta = 0.67, 95% CI =  − 0.101—1.434, *p* = 0.09).

## Discussion

This study aimed to assess the impact of different Bengali translation approaches for SACT patient information booklets and consent forms. In the Booklet Component, we observed a higher rate of critical errors introduced by machine translation compared to professional translation, but that only 16% of participants across both translation arms correctly understood treatment intent after reading. In the Consent Component, we observed significantly higher rates of both measured comprehension of treatment intent and subjective confidence in understanding, in participants receiving a bilingual consent form.

The role of unsupervised machine translation in healthcare remains contentious. Without human review, machine translations can create clinical risks, which vary by language [17]. A recent study comparing neural machine translation tools to professional translations in the translation of discharge summaries showed equivalent quality for Spanish (a high-resource language), but inferior quality for Chinese, Vietnamese and Somali translations (lower resource languages) [20]. Our results support caution in the use of unsupervised machine translation tools, especially in low-resource languages such as Bengali. If healthcare organisations do provide unsupervised machine translations through accessibility tools, they should be aware of risks and should provide disclaimers in the target language [32]. Producers of patient information can also maximise “translation-friendliness”, for example by avoiding idioms and complex sentences, acknowledging that other users may access the material via machine translation [32]. Many computational linguists and clinical researchers suggest that the next step for machine translation (including large language models) should be human-centred machine translation [33].

Despite the clear difference in translation quality in the two arms of the Booklet Component, we observed a low level of comprehension in participants after reading translated written patient information, whichever translation method (machine or professional) they were randomised to. This is despite choosing a booklet designed for accessibility and readability, suggesting that low health literacy or other communication factors may be additional barriers beyond the availability of translated resources. Other formats of patient information, such as audio and video, may be more effective in some linguistic groups and deserve further study.

Participants’ understanding of treatment intent improved significantly in both arms of the Consent Component compared to the Booklet Component, likely reflecting cumulative learning and the additional benefit of an interpreted verbal consent consultation. The improvement was substantially greater in the group receiving the English–Bengali bilingual SACT consent forms, who demonstrated significantly higher comprehension and confidence than participants provided with English-only forms. Two mechanisms may explain the improved comprehension and confidence seen with the bilingual consent form. First, in addition to hearing the interpreted consultation, participants could directly read the information in Bengali. Second, interpreters could read aloud Bengali translations of specialist terminology, rather than interpreting these terms spontaneously. This latter benefit may be even more marked if less experienced interpreters are interpreting treatment consent consultations. However, the combination of written translation and verbal interpretation was not universally effective: within the bilingual consent form group, 40% of participants did not correctly understand the intent of treatment. Further investigations are necessary to ascertain whether using accessible spoken Bengali, normally understood by Sylheti speakers, contributed to the limited understanding of the intent of treatment for the bilingual consent group.

Our study has several limitations. First, we recruited healthy volunteers rather than patients with cancer. Patients face additional psychological and cognitive demands which we cannot account for in our study, though such burdens would likely make reliable translations even more valuable. The salience of treatment intent would also be greater in a patient with cancer than a healthy volunteer. Second, our simulation had partial fidelity to real-life clinical practice, with small groups of participants undertaking consent consultations rather than individuals. Third, we measured comprehension and confidence immediately after each intervention, rather than looking at recall later. Fourth, we did not include a formal measurement of health literacy in Bengali [34]; this limits our ability to assess the contribution of health literacy to our study endpoints. Fifth, our study was exploratory and not definitively powered to our objectives, resulting in wide confidence intervals for effect sizes. Finally, machine translation of Bengali is likely to perform worse compared to languages where machine translation tools have had a larger linguistic corpus for training (e.g. French, Spanish, German), and poor performance of machine translation may not apply across other language groups.

We recommend adopting bilingual interlinear consent forms as a cost-effective and relatively simple measure to improve communication and health equity for LEP patients. Bilingual consent forms may be effective in other language-discordant health contexts beyond SACT, including clinical trials, especially when personalisation can be achieved without writing extensive text (e.g. through ticking relevant boxes). Further work is needed to assess the role of machine translation in healthcare communication, but current use should be cautious. Finally, other methods beyond written booklets should be explored to provide health information for LEP patients.

## Supplementary information

Below is the link to the electronic supplementary material.

ESM 1 (DOCX 31.9 KB)

ESM 2 (DOCX 429 KB)

## Data Availability

The dataset generated and analysed during the current study are available in the Zenodo repository, doi: 10.5281/zenodo.17580808. The statistical analysis plan of the study was registered in the Open Science Framework, [https://www.osf.io/axg5d](https://www.osf.io/axg5d).
