# Supplementary material for: Translation approaches to support systemic anti-cancer therapy consent for individuals with limited English proficiency
Source: Support Care Cancer. 2026 Mar 13;34(4):317. doi: 10.1007/s00520-026-10464-w (PMC12987779; doi:10.1007/s00520-026-10464-w)
Supplement: Supplementary file 2 — (DOCX 429 KB) [file 520_2026_10464_MOESM2_ESM.docx]

**Supplementary Content**

**Supplementary Methods**: Additional detail for study interventions; Additional detail for study outcomes; Patient and public involvement (PPI) in study design

**Supplementary Results:** Full reports of independent translation assessments

**Supplementary References**

**Supplementary Table 1**: Summary demographic information of included participants

**Supplementary Table 2:** Summary of primary outcomes by randomisation status

**Supplementary Figure 1**: Booklet Component primary outcome associations with demographic variables and randomisation status

**Supplementary Figure 2:** Histogram of Consent Component Total Comprehension Score

# Supplementary Methods

## Additional detail for study interventions

#### Booklet Component:

The “Easy Read: How Is Myeloma Treated?” booklet was written by the patient charity Myeloma UK and is used in UK clinical contexts.^1^ The booklet is designed to provide accessible and easy-to-read information, and is written in accordance with the European Easy-to-Read standard. The aim of the standard is primarily to produce information that is accessible to people with learning disabilities, but documents produced to the standard may also be helpful to others, such as those with LEP. Like most patient information booklets, the “Easy Read: How Is Myeloma Treated?” booklet was not primarily designed as a resource to be translated, but during production of patient information, consideration is given to “translation-friendliness”, such as avoiding idioms and complex tenses.

We created two translations of this booklet. For the machine translation, we asked Bengali translators to recommend a suitable neural machine translation tool for the low-resource English into Bengali language combination, and chose Google Translate, which has been widely tested in healthcare settings. A research team member who cannot read Bengali used Google Translate to create the machine translation, preserving the original booklet format as best possible.

The professional translation was commissioned to professional translators and completed according to International Organization for Standardization (ISO) standards (ISO 17100:2015+A1:2017 – *Translation services – Requirements for translation services*). In this process, one translator completes the translation and their work is independently revised by another translator, who checks the Bengali translation alongside the English original. Finally, a Bengali-speaking proofreader reads the translation as if it had been originally written in Bengali. Any use of machine translation renderings by the first translator was post-edited respecting the requirements of ISO 18587:2017 (*Translation services. Post-editing of machine translation output*).

Participants were recruited from local Bengali and Sylheti speaking community groups and met in community venues in groups of between 12-40 participants across five sessions. Sessions were led by English and Bengali-speaking researchers, with an accredited Bengali and Sylheti interpreter present at each session. Participants first watched an introductory video in Bengali, which explained that they would be asked to imagine that they were supporting an imaginary friend or family member who had just been diagnosed with myeloma, and who was now asking for their assistance in understanding an information booklet they had been given.

After written consent was collected, participants were given a sealed envelope containing their allocated group (“circle” or “square” to maintain blinding), allocated booklet translation, a Bengali form to collect demographic information, and Booklet Comprehension Tool. The two groups were taken to separate physical spaces (different rooms or parts of a room). After completing their demographic information, participants were given 15 minutes to read the translated booklet.

After the allocated reading time, research team members asked participants to put away their booklets, and take out the Booklet Comprehension Tool. The interpreter read each question and each potential answer out loud. Any participant asking for help with an answer could have the question repeated, but was not given guidance to select a specific answer. After collection of the completed Booklet Comprehension Tool, participants were given the opportunity to ask any questions they had about myeloma and myeloma treatment. All participants were given a £20 gift voucher for participation in the Booklet Component and invited to return for the Consent Component.

Otherwise eligible friends and contacts of participants, who had been unable to attend the Booklet Component, were allowed to attend the Consent Component. To reflect the difference in this group, we organised a separate randomisation sequence for participants attending the Consent Component who had not participated in the Booklet Component. However, this simulated consent-only cohort was small (n=29) and did not meet our pre-specified size for analysis (>40); this cohort is not described further within this paper.

#### Consent Component

Haemato-oncologists (qualified doctors with at least five years of postgraduate training) with experience in taking consent for SACT were randomly assigned to either the English-only or bilingual consent form groups, taking place in different rooms. Each doctor was supported by a Bengali/Sylheti interpreter accredited as a member of the National Register of Public Service Interpreters (NRPSI) in the UK. Full NRPSI accreditation is awarded to interpreters who possess professional qualifications recognised by the National Occupational Standards for Interpreting (e.g., the exam-based Diploma in Public Service Interpreting (DPSI) from the Chartered Institute of Linguists) and have completed over 400 hours of active interpreting practice.

The UK Generic SACT Form is hosted by Cancer Research UK and approved by the UK National SACT Board. It is approved for use in consent conversations in hospitals across the UK. The current form is available in English and Welsh only.^2^ We commissioned a gold-standard Bengali translation of this form meeting the requirements of both ISO 17100:2015+A1:2017. *Translation services – Requirements for translation services* and 18587:2017. *Translation services. Post-editing of machine translation*. We edited this translation to preserve an interlinear format.

Individuals returning for the Consent Component were given a second sealed envelope containing their allocated room (“red room” or “green room” to maintain blinding). Depending on the number of individuals attending at any given time, groups of 4-12 individuals were brought through at a time to their allocated room. They were again asked to imagine that their imaginary friend or family member had asked them to attend a hospital appointment where treatment would be discussed.

Using the SACT consent form for structure, the doctor conducted a SACT consent consultation to talk through the benefits, practicalities and risks of daratumumab, bortezomib, thalidomide and dexamethasone with the aid of the interpreter. Following this explanation, the doctor and interpreter left the room, and a Bengali/Sylheti-speaking researcher read out each question in the Consent Comprehension Tool and invited participants to complete this. Any participant asking for help with an answer could have the question repeated, but was not given guidance to select a specific answer. All participants were given a £20 gift voucher for participation in the Consent Component.

## Additional detail for study outcomes

Comprehension assessments were devised through several stages. Firstly, research team members decided on the primary outcome of understanding treatment intent, and sought consensus for other points of information that should be included as critical for valid consent for a patient commencing myeloma therapy.

To identify these, we undertook a prioritisation exercise through the UK Myeloma Research Alliance. Between April and June 2024, we received responses from seven haemato-oncology consultants and five cancer nurse specialists. We also met with three Bengali- or Sylheti- speaking myeloma patients and family members to ask their experience of providing informed consent.

Using these responses, we designed two comprehension tools (Booklet Comprehension Tool and Consent Comprehension Tool), designed to assess both the primary outcome and a Total Comprehension Score (comprised of other pieces of information deemed critical for consent), after completion of each component. We prompted the large language model Microsoft 365 Copilot Version 2407 (Build 17830.20128), based on GPT-4 (Open-AI), asking for suggested simplification of the language used in these draft questions, and edited the outputs. Clinicians in the research team evaluated the questions in English for clarity of formulation and accuracy.

Next, we shared the edited simplified questions with a patient and public involvement sounding board, which included an individual with experience of myeloma. With this group, we conducted a comprehension validation session of the Comprehension Tools in English, with native speakers of English. Validation participants suggested refinements and clarifications. We incorporated these for the final comprehension tools in English. Then, we commissioned professional translations of these refined comprehension tools into Bengali, which were scrutinised for accuracy and suitability by the Bengali-speaking clinicians in the research team.

### Definition of primary outcome for Booklet Component

A participant was classified as correctly understanding treatment intent if they provided correct answers to **both** of the following questions in the Booklet Comprehension Tool (correct answers identified in bold):

What is the main goal of the treatment given to treat myeloma?

- Cure myeloma completely for everyone.
- Cure myeloma in most patients, but not everyone will be cured.
- **Make the myeloma go away for a while to help people live longer and feel better, but not cure it.**
- None of the above.

The medicines for myeloma can cure the disease completely.

- True
- **False**

### Definitions of secondary outcomes for Booklet Component

*Definition of Booklet Component Total Comprehension Score*

A participant received one mark for each correct answer to each of the questions comprising the primary outcome (above) in addition to one mark for each correct answer to the questions below (correct answer designated in bold). The maximum possible score is 5.

What does “remission” mean in the context of myeloma treatment?

- When myeloma is completely cured.
- **When myeloma goes away for a time after treatment, but it will come back in future.**
- When myeloma treatment does not work.
- When myeloma comes back after treatment.

What should a patient do if they are unsure about when to take their medicines?

- Wait until their next hospital visit.
- Ask a friend.
- **Speak to their doctor or nurse.**
- Look it up online.

Remission means that myeloma has been cured forever.

- True
- **False**

#### Definition of Confidence in explaining myeloma to a family member

After answering the comprehension questions in the Booklet Comprehension Tool (as detailed above), participants were asked the question “How confident would you feel to explain information in this booklet to your imaginary friend or family member?”, and could choose one of five discrete responses: “Not Confident at All”, “Slightly Confident”, “Moderately Confident”, “Very Confident” or “Extremely Confident”.

#### Definition of Perceived clarity of language

Following the “*Confidence in explaining myeloma to a family member*” question, participants were asked to choose a response to “How clear was the language in the booklet?”. Discrete options provided were “Not Clear at All”, “Slightly Clear”, “Moderately Clear”, “Very Clear” or “Extremely Clear”.

### Definition of primary outcome for Consent Component

A participant was classified as correctly understanding treatment intent if they provided correct answers to **both** of the following questions in the Consent Comprehension Tool (correct answers identified in bold):

What is the main goal of the treatment given to treat myeloma?

- Cure myeloma completely for everyone.
- Cure myeloma in most patients, but not everyone will be cured.
- **Make the myeloma go away for a while to help people live longer and feel better, but not cure it.**
- None of the above.

Will this type of myeloma treatment cure them?

- Everyone is cured by this treatment.
- Most people are cured by this treatment.
- Some people are cured by this treatment.
- **Nobody is cured by this treatment, but it can make the myeloma go away for a while to help people live longer and feel better.**

### Definitions of secondary outcomes for Consent Component

#### Definition of Consent Component Total Comprehension Score

A participant received one mark for each correct answer to each of the questions comprising the primary outcome (above) in addition to one mark for each correct answer to the questions below (correct answer designated in bold). The maximum possible score is 6.

How is myeloma treatment given?

- The patient stays in the hospital for one week each month and gets medicine every day.
- The patient takes all their medicine as tablets at home.
- **The patient visits the hospital each week for some medicine and takes other medicine as tablets at home.**
- None of the above.
- I cannot remember.

Which of these statements are true about pregnancy while on myeloma treatment?

- Both women and men can safely conceive a pregnancy while on myeloma treatment.
- Women should not get pregnant, but men can safely get their partner pregnant while on treatment.
- People on myeloma treatment cannot have babies because they will be infertile.
- **It’s unsafe to conceive a pregnancy for anyone on myeloma treatment.**

Which statement is TRUE?

- People with myeloma cannot safely have a blood transfusion.
- **Some patients receiving myeloma treatment need blood transfusions to help with anaemia (low red blood cell levels).**
- The information is not provided.

What should they do if they develop a fever?

- This is normal with the treatment, and they should take paracetamol.
- They should talk to their general practitioner when they can.
- They should talk to their haematology consultant at their next clinic appointment.
- **This is an emergency, and they need to go to the hospital right away.**

#### Definition of Self-rated confidence in understanding

Following the comprehension questions in the Consent Comprehension Tool (as detailed above), participants were asked two separate questions to assess their confidence: “After hearing this discussion, how confident are you in knowing what to do if your friend or family member has questions about their myeloma treatment?” and “How confident do you feel that you understood the main points of the discussion?”. Participants could select one of five discrete responses for each question: “Not Confident at All”, “Slightly Confident”, “Moderately Confident”, “Very Confident” or “Extremely Confident”.

# Patient and public involvement (PPI) in study design

We report PPI in this study using the suggested items in the Guidance for Reporting Involvement of Patients and the Public (GRIPP2) short form.^3^

The aims of PPI in this study were to a) identify outcomes of importance to Bengali- or Sylheti- speaking people with LEP living with myeloma, b) to assess suitability of our planned interventions and comparators, and c) to improve acceptability of our study design for potential participants.

We presented the study design to British-Bangladeshi community leaders across several meetings, both live and virtually, and incorporated to their feedback and suggestions. In addition, we met with two Bengali- speaking individuals with lived experience of myeloma and one family member of a Bengali-speaking individual with myeloma, to listen to their experiences of myeloma diagnosis and SACT consent, and how this related to translation needs. We shared our draft translated study documents with this group to seek feedback on how they would have experienced these at the time of their diagnosis. Finally, we piloted our study design on a small PPI group, including an individual with lived experience of myeloma.

As a result of these meetings, we refined our study outcomes to incorporate subjective confidence in understanding as well as testable comprehension. Community leaders identified suitable community groups for recruitment, highlighted the importance of single-sex spaces, and directed us away from research activities on Friday (an important day for Islamic prayers – the majority of potential participants were Muslim).

During the meeting with Bengali-speaking people with lived experience of myeloma and LEP, the interpreter pointed out the value of the bilingual consent form for their *interpretation* as well as for the patients to read, giving insight into additional value of the translated form. Even though our research team comprised multiple Bengali- and Sylheti-speaking members, professional interpretation and translation were necessary for both PPI and research activities. As a result, we experienced the cost implications of both interpretation and translation during the research project, helping us reflect on the financial and logistic challenges faced by healthcare organisations and patient charities.

**Supplementary Results**

# Blinded, independent assessment of machine translated booklet

Total score: 51/100

Aspect 1: Comprehension, Accuracy, Transfer of names, dates and figures, lexis

Aspect 1 Score: 23/50

A reasonable level of comprehension was demonstrated throughout the document. However, in several instances, there were serious issues with comprehension, resulting in text that is completely inaccurate.

For example, the sentence, *The medicine will help you to: Kill as many myeloma cells as possible,* was mistranslated as instructing the patient to kill as many myeloma cells as possible. Similar issues were repeated multiple times in other parts of the text.

Another example of a comprehension issue is the sentence, *We can put you in touch with another myeloma patient to talk to,* which was mistranslated into an incoherent Bengali sentence that roughly translates to: "We can contact other patients for us to talk to you."

These examples illustrate significant errors that impact the accuracy and clarity of the translation, requiring careful revision to meet professional standards.

Most of the document was translated with a satisfactory level of accuracy.

However, in several instances, as outlined in the comprehension feedback above, the translated text conveys a completely different meaning from the source text and is often

incoherent.

For example:

• Peer support was simply translated as "Peer" without conveying the full meaning.

• About Myeloma UK was translated as "Related Myeloma UK" which does not make sense in Bengali.

• Side effects was translated as "Harmful side" despite there being an exact and commonly used Bengali equivalent for this term.

These errors significantly affect the accuracy and clarity of the translation, indicating a need for careful revision.

Aspect 2: Quality of the Target Language (grammar, syntax, cohesion, coherence, organisation of work, orthography, punctuation, accentuation, register)

Aspect 2 Score: 28/50

Most of the translated text maintains a satisfactory level of grammar and syntax. However, in several instances, the sentences are incoherent, unnatural, and difficult to understand due to syntax or grammatical issues.

One example is the translation of the sentence, *You might get more infections,* which was translated in a way that made the Bengali sentence unnatural and incomprehensible due to both syntax and grammar problems.

A similar issue occurs with the sentence, *Talk to your doctor or nurse if you are worried or have any questions about your treatment,* which also suffers from unnatural phrasing and lack of clarity in the translation.

These issues highlight the need for careful attention to grammar and syntax to ensure the translation is both accurate and easy to understand.

The text overall maintains a satisfactory level. However, there are numerous instances of both minor and major issues that change the meaning and make the text difficult to follow.

For example, there are some minor spelling errors, such as
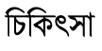
 (*treatment*) often being spelled as
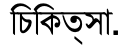
. While this is considered a minor issue because the incorrectly spelled word can still be recognised and read as the intended word, in some cases, misspellings can alter the meaning. For instance, the word *"any"* was translated as (
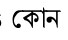
 (kon), meaning *"which"*, instead of the correct
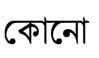
 (kono) or
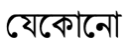
 (jekono).

In addition to the examples provided in the sections above, another example of an incoherent sentence due to grammatical issues is the translation of the sentence: *At the hospital you will be looked after by a special doctor and nurse.* The translation reads as though the nurse would be looked after, which misrepresents the intended meaning.

These errors, particularly those that lead to changes in meaning or incoherence, significantly impact the quality of the translation and highlight areas requiring careful revision.

# Blinded, independent assessment of professionally translated booklet

Total score: 73/100

Aspect 1: Comprehension, Accuracy, Transfer of names, dates and figures, lexis

Aspect 1 Score: 33/50

Overall, the quality of the translation is good, demonstrating good comprehension. The subject matter was well understood, and the meanings were conveyed accurately most of the time.

However, there are a few instances where complete comprehension was not achieved. For example, *Help you live longer* was translated as “Help you live long” which implies that the person will have a long life. This is not what the source text conveys. The source text indicates that the medicine will help the person live a longer life compared to what it would have been without the medicine.

Similarly, *You might not be able to sleep* was translated as "Your sleep might be disrupted" which describes two different situations.

Overall, the text maintains a good standard of accuracy. With minor corrections, it could reach a professionally acceptable level.

For example, the word *infections* is bold in the source text but not in the translated text. In this easy-to-read version, it is very important for *infections* to be bold to maintain emphasis and readability.

Additionally, in the sentence, *Your doctor will tell you which medicines to take and when to take them,* the words *to take* were omitted in the translated text, making it sound slightly unnatural and difficult to understand.

Aspect 2: Quality of the Target Language (grammar, syntax, cohesion, coherence, organisation of work, orthography, punctuation, accentuation, register)

Aspect 2 Score: 40/50

Overall, the translation is written in a good standard of Bengali. However, in some instances, a few sentences are slightly difficult to understand due to issues with syntax. For example, the meaning of the sentence, *We work with others to find, develop and give patients new myeloma treatments*, is not entirely clear in the translated text upon first reading. This suggests that the structure or phrasing could be improved for better clarity and flow.

The text overall maintains a good level of clarity and orthography, although some minor spelling and grammar issues could be addressed to improve the readability and accuracy of the translated text.

For example, the plural Bengali word "
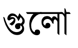
 (gulo)" was consistently written as "
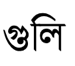
 (guli)" throughout the text. "
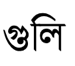
(guli)" is associated with Sadhu Bhasa, an older form of Bengali, which is less appropriate for modern, easy-to-read texts.

Additionally, in the sentence *You can read this booklet with a friend or a doctor or nurse and talk about it with them*, the word *nurse* was translated with incorrect grammar, making the sentence difficult to understand. These issues highlight areas for refinement to enhance the overall quality of the translation.

# Supplementary References

1. Myeloma UK. Easy Read: How is myeloma treated? <https://www.myeloma.org.uk/library/easy-read-how-is-myeloma-treated/>. Accessed 11 November 2025.
2. Cancer Research UK. Consent forms for SACT (Systemic Anti-Cancer Therapy). Cancer Research UK. September 9, 2016. <https://www.cancerresearchuk.org/health-professional/treatment-and-other-post-diagnosis-issues/consent-forms-for-sact-systemic-anti-cancer-therapy>. Accessed 11 November 2025.
3. Staniszewska S, Brett J, Simera I, et al. GRIPP2 reporting checklists: tools to improve reporting of patient and public involvement in research. BMJ. 2017;358:j3453; doi:10.1136/bmj.j3453

# Supplementary Tables and Figures

**Supplementary Table 1:** Summary demographic information of included participants, stratified by randomisation status in both Booklet Component and Consent Component. Values are % of participants

|  |  |  | **Booklet Randomisation** | |  | **Consent Randomisation** | |
| --- | --- | --- | --- | --- | --- | --- | --- |
| **Variable** | **Response** | **All Booklet Component** | **Machine Translation** | **Professional Translation** | **All Consent Component** | **English-only consent form** | **Bilingual consent form** |
| **N** |  | **121** | **61** | **60** | **91** | **46** | **45** |
| Gender | Male | 57.9% | 57.4% | 58.3% | 53.3% | 58.7% | 47.7% |
|  | Female | 42.1% | 42.6% | 41.7% | 46.7% | 41.3% | 52.3% |
| Age | 18-24 | 5.8% | 6.6% | 5.0% | 4.4% | 4.3% | 4.5% |
|  | 25-34 | 7.4% | 4.9% | 10.0% | 10.0% | 8.7% | 11.4% |
|  | 35-44 | 19.8% | 19.7% | 20.0% | 18.9% | 10.9% | 27.3% |
|  | 45-54 | 28.9% | 36.1% | 21.7% | 32.2% | 34.8% | 29.5% |
|  | 55-64 | 36.4% | 31.1% | 41.7% | 32.2% | 37.0% | 27.3% |
|  | 65 and over | 0.8% | 0.0% | 1.7% | 1.1% | 2.2% | 0.0% |
|  | Prefer not to say | 0.8% | 1.6% | 0.0% | 1.1% | 2.2% | 0.0% |
| What is the highest level of education you have completed? | No response | 9.1% | 11.5% | 6.7% | 6.7% | 6.5% | 6.8% |
|  | No formal education | 5.0% | 8.2% | 1.7% | 4.4% | 2.2% | 6.8% |
|  | Primary education | 28.1% | 31.1% | 25.0% | 31.1% | 32.6% | 29.5% |
|  | Secondary education | 37.2% | 29.5% | 45.0% | 35.6% | 37.0% | 34.1% |
|  | Vocational qualification | 2.5% | 0.0% | 5.0% | 3.3% | 2.2% | 4.5% |
|  | Bachelor’s degree | 7.4% | 6.6% | 8.3% | 6.7% | 4.3% | 9.1% |
|  | Master’s degree | 5.8% | 8.2% | 3.3% | 6.7% | 6.5% | 6.8% |
|  | Doctorate | 1.7% | 1.6% | 1.7% | 1.1% | 2.2% | 0.0% |
|  | Prefer not to say | 3.3% | 3.3% | 3.3% | 4.4% | 6.5% | 2.3% |
| How would you rate your ability to use English? | No response | 2.5% | 1.6% | 3.3% | 2.2% | 2.2% | 2.3% |
|  | Very poor | 13.2% | 16.4% | 10.0% | 15.6% | 19.6% | 11.4% |
|  | Poor | 9.9% | 9.8% | 10.0% | 10.0% | 8.7% | 11.4% |
|  | Fair | 52.9% | 49.2% | 56.7% | 52.2% | 47.8% | 56.8% |
|  | Good | 14.0% | 14.8% | 13.3% | 13.3% | 17.4% | 9.1% |
|  | Very good | 2.5% | 3.3% | 1.7% | 2.2% | 2.2% | 2.3% |
|  | Excellent | 5.0% | 4.9% | 5.0% | 4.4% | 2.2% | 6.8% |
| How confident are you in reading and understanding booklets in English? | No response | 3.3% | 4.9% | 1.7% | 2.2% | 4.3% | 0.0% |
|  | Not confident at all | 24.0% | 24.6% | 23.3% | 24.4% | 23.9% | 25.0% |
|  | Slightly confident | 24.8% | 23.0% | 26.7% | 25.6% | 17.4% | 34.1% |
|  | Moderately confident | 30.6% | 27.9% | 33.3% | 32.2% | 37.0% | 27.3% |
|  | Very confident | 13.2% | 14.8% | 11.7% | 12.2% | 15.2% | 9.1% |
|  | Extremely confident | 4.1% | 4.9% | 3.3% | 3.3% | 2.2% | 4.5% |
| What do you know about myeloma? | No response | 5.0% | 6.6% | 3.3% | 3.3% | 4.3% | 2.3% |
|  | I do not know what myeloma is. | 80.2% | 73.8% | 86.7% | 82.2% | 82.6% | 81.8% |
|  | I know a little bit about myeloma. | 9.9% | 13.1% | 6.7% | 10.0% | 8.7% | 11.4% |
|  | I know a moderate amount about myeloma. | 4.1% | 4.9% | 3.3% | 4.4% | 4.3% | 4.5% |
|  | I know a large amount about myeloma. | 0.8% | 1.6% | 0.0% | 0.0% | 0.0% | 0.0% |

**Supplementary Table 2:** Summary of primary outcomes by randomisation status. Odds ratios were calculated from univariate and multivariate regression models - the latter were adjusted for all demographic characteristics captured in Supplementary Table 1. Additionally, the Consent Component multivariate model was adjusted for Booklet Component randomisation status, because the Consent Component took place after the Booklet Component, with the same participants included.

|  |  | | **Univariate** | | | | **Multivariate** | | | |
| --- | --- | --- | --- | --- | --- | --- | --- | --- | --- | --- |
| **Outcome** | **Exposure** | **N** | **OR** | **CIU** | **CIL** | **P** | **OR** | **CIU** | **CIL** | **P** |
| Booklet Component Primary outcome | Booklet Randomisation | 121 | 1.154684 | 3.074114 | 0.433717 | 0.77343 | 0.989736 | 2.993242 | 0.327263 | 0.985422 |
| Consent Component Primary outcome | Consent Randomisation | 91 | 2.8125 | 6.585509 | 1.201146 | 0.0172 | 3.733396 | 10.13592 | 1.375134 | 0.009734 |

**Supplementary Figure 1**: Booklet component primary outcome associations with demographic variables and randomisation status


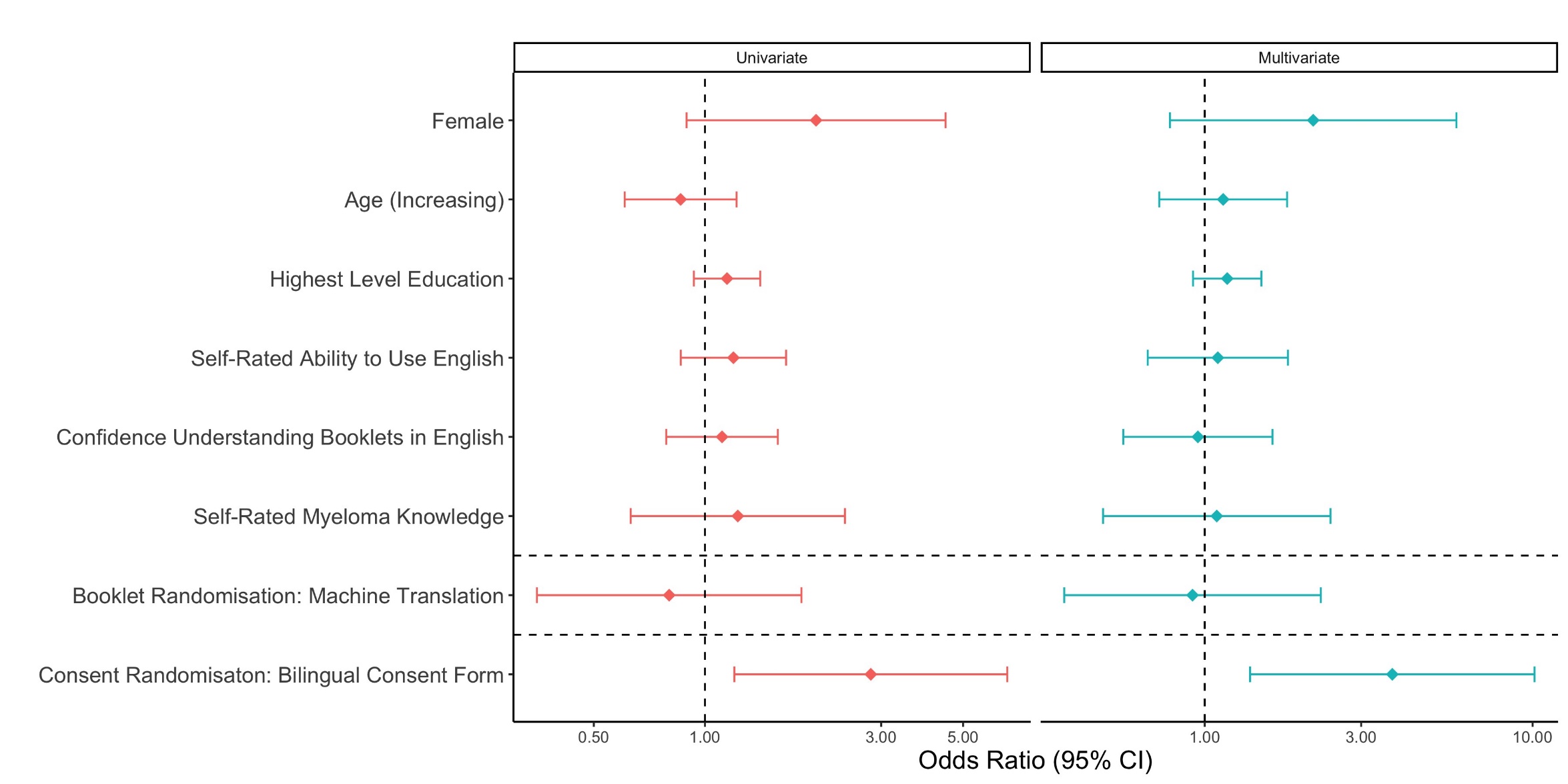


**Supplementary Figure 2:** Histogram of Consent Component Total Comprehension Score. A score of 0 represents no correct answers, and 6 represents all questions correctly answered. Top panel: score distribution for participants randomised to the bilingual consent form. Bottom panel: score distribution for participants randomised to the English-only consent form.

**
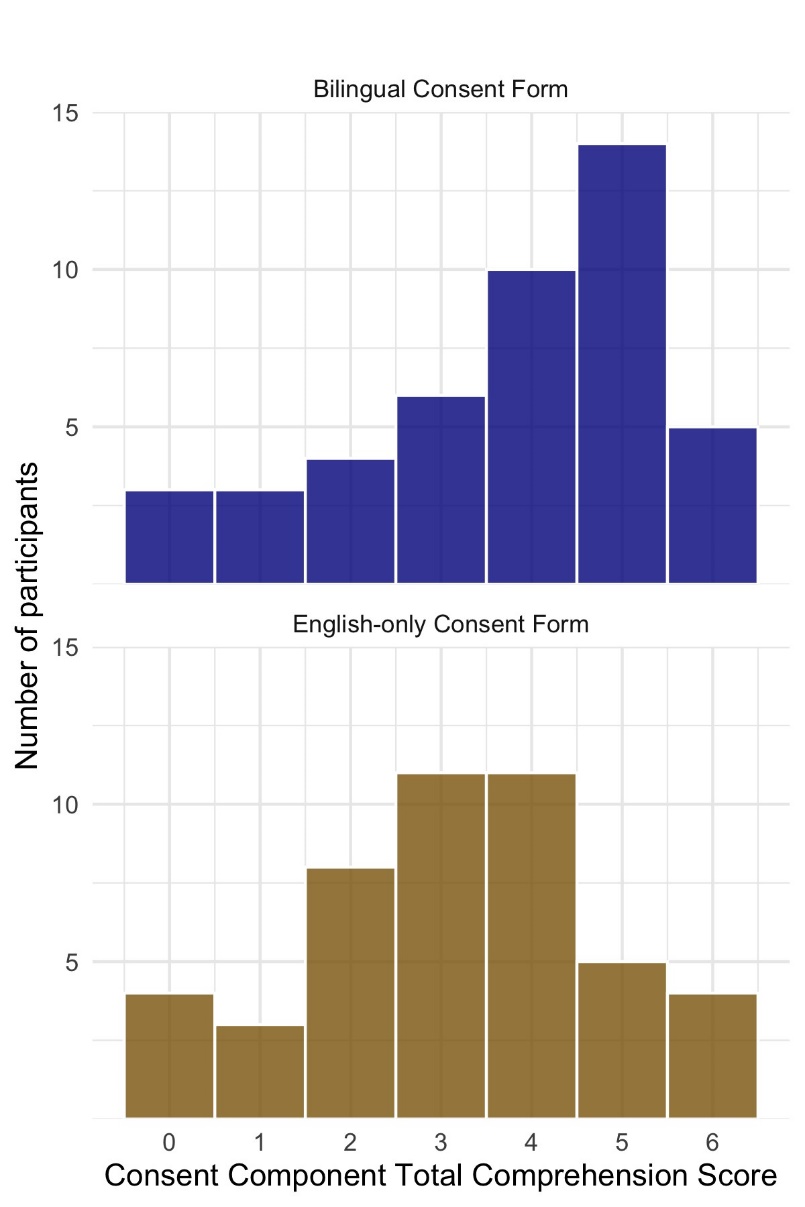
**
